# Improving students' performance via case-based e-learning

**DOI:** 10.3389/fmed.2024.1401124

**Published:** 2025-01-06

**Authors:** Sebastian Ertl, Patricia P. Wadowski, Henriette Löffler-Stastka

**Affiliations:** ^1^Department of Psychoanalysis and Psychotherapy, Medical University of Vienna, Vienna, Austria; ^2^Division of Internal Medicine II (Cardiology and Intensive Care Medicine, Angiology) Ordensklinikum Linz, Linz, Upper Austria, Austria; ^3^Department of Internal Medicine II, Division of Angiology, Medical University of Vienna, Vienna, Austria

**Keywords:** case-based blended learning, computer-assisted instruction, e-learning, distance learning competence, transfer learning

## Abstract

**Background:**

The integration of interdisciplinary clinical reasoning and decision-making into the medical curriculum is imperative. Novel, high-quality e-learning environments, encompassing virtual clinical and hands-on training, are essential. Consequently, we evaluated the efficacy of a case-based e-learning approach.

**Method:**

The impact of a case-based interdisciplinary e-learning environment with an integrated question/feedback system on medical students' academic performance was examined in a cross-sectional sample of different study years, longitudinally matched per academic year. Differences between the groups were analyzed through an unpaired *t*-test.

**Results:**

The exam results of students who participated in the e-learning course (*n* = 296) were compared with those of all students at the university (*n* = 5,800). A statistically significant (*p*-value < 0.01) improvement in students' final examination grades was observed through intensive training on the offered platform.

**Conclusions:**

Our analyses demonstrated the positive influence of a case-based e-learning approach within the Viennese medical curriculum.

## 1 Introduction

Clinical reasoning is an essential tool for all kinds of thinking processes. It consists of developing internalized terms [e.g., via scenarios or integrated thematic instructions (1)] and their integration into new situations. Clinical reasoning can be divided into intuitive and analytical components (2). Research on mental processes shows that key characteristics for training the analytic components are disease patterns stored in 'frames', clinical scenarios, semantic networks or qualifiers, or illness scripts. Repeated presentation and exercise of clinical cases are crucial for efficient learning (2–4).

Associative learning must be stimulated to provide a training tool that helps students transfer their declarative knowledge to procedural knowledge. In case of associative learning, two stimuli have a temporal relationship: a person responds to the first stimulus in anticipation of the second (neural link/association = foundation) (5–8). As a result of the reclassification of stimuli, response drivers arise: conditional appetence, conditional action, conditional aversion, and conditioned inhibition. For clinical reasoning trained through associative learning, procedural knowledge must be built through polythematic or crosslinking thinking. The ability to link information (thoughts, symbols, images, and scenes) in a meaningful way and master it requires creative processes that combine seemingly unrelated areas (domains) (9–14). Case-based learning environments are optimal for teaching and training clinical reasoning (7).

To foster motivational aspects of lifelong learning in students, e-learning is an ideal approach for teaching students (15). Moreover, controlled e-learning programs with detailed feedback are a tool to prevent emotional exhaustion and are highly accepted among students (16). As one form of e-learning, distance learning is defined by UNESCO as the separation of teachers and learners of various shapes in space or time in exchange for electronic or printed materials (15, 17).

Martin et al. (18) evaluated the term' online learning' to specify the latest form of distance education. Significant developments have occurred since the first flare-up on the horizon of didactics, which have also been scientifically supported. Over time, meta-analyses and second-order meta-analyses show the significant superiority of distance learning over face-to-face learning (18). One aspect that fosters learning efficacy is affective involvement (13, 19). Affective involvement, mentalized affectivity, the intuitive and analytical components (c.f. slow and fast thinking processes) are the basis and target factors to be addressed in training (20). Especially for clinical reasoning the importance of associative learning is evident; the stimulus-response principle needed in clinical reasoning is best trained via high affective involvement, time-space autonomy, which is predominantly provided in e-learning formats.

In terms of e-learning, newer concepts based on artificial intelligence and modern visualization software have become, of course, effective teaching methods (21), especially when they are constructed playfully; previous analysis showed good to high acceptance and satisfaction with the course and learning method (13, 22, 23) and had shown to be a bridge to research, especially fostering translational research (10).

AI-supported systems were recently applied and evaluated in a pilot online simulation training based on the dual process theory of clinical reasoning, that is a practicum script, which showed alignment with neurocognitive science principles and a high acceptance among students (24). However, this method lacks interaction with simulated patient training, and outcomes like improved student grades have not yet been evaluated (24).

Hornos et al. stated that errors in complex and uncertain situations are not triggered by inadequate knowledge but by cognitive failures (24). At a certain level, medical students should be trained by patient cases to simulate clinical scenarios over theoretical knowledge (24, 25). Simulation platforms become increasingly popular and show outstanding results (18, 24). It is our conviction that scientific support and the inclusion of scientific professional societies and specialist associations for each medical specialty are essential.

### 1.1 Purpose of the study and research question

This study aimed to demonstrate how a case-based e-learning approach influences medical students' grades. We hypothesized that the average exam results would improve with additional training with our case-based elective course compared to the students without any extra training. However, the superiority of case-based teaching can only be proven by a hard endpoint, such as the final grade.

The study findings should facilitate a discussion on the role of a case-based e-learning approach as an essential part of undergraduate and postgraduate education. Certainly, case-based teaching can be implemented without e-learning; however, this aspect was not examined in the current study and will be addressed in subsequent research.

Furthermore, the results of this study should contribute to the enhancement of e-learning methodologies, as the teaching methods incorporate a feedback system and authentic clinical scenarios (22).

## 2 Methods

### 2.1 Development of the training course

**Figure 1:**
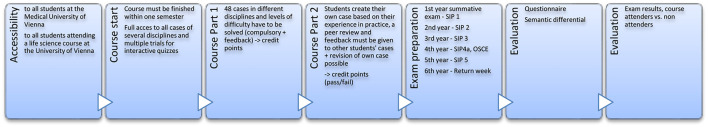
Course description, timeline, task definition, and assignments.

In 2016, the course “Fallorientierte Lehre (Case-based teaching)—Übungen (Practice): Clinical Reasoning and Clinical Decision Making” was introduced at the Medical University of Vienna provided on a Moodle e-Learning platform (26) (Figure 1).

The course is the result of extensive research in the field of case-based learning. Patient histories from individuals treated at the General Hospital of Vienna were anonymized and processed to learning material, starting in 2014 (27). Guidelines and quizzes have been incorporated into this learning material to create patient cases (25). The test sections were analyzed to ensure good quality and efficacy (28).

**Figure 2:**
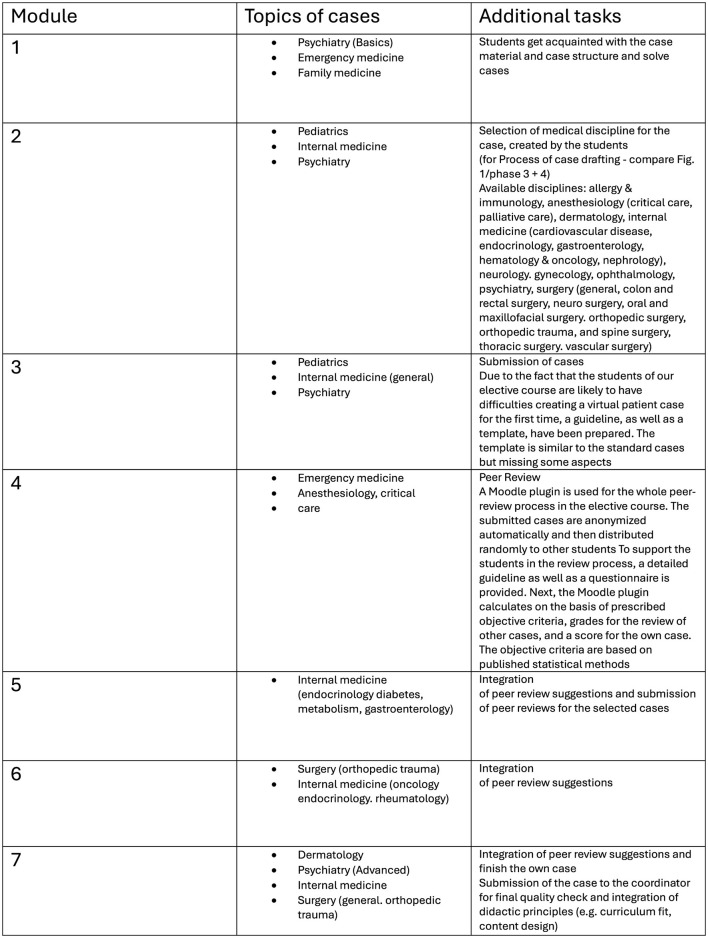
Course structure adapted from Ertl et al. (13).

**Figure 3:**
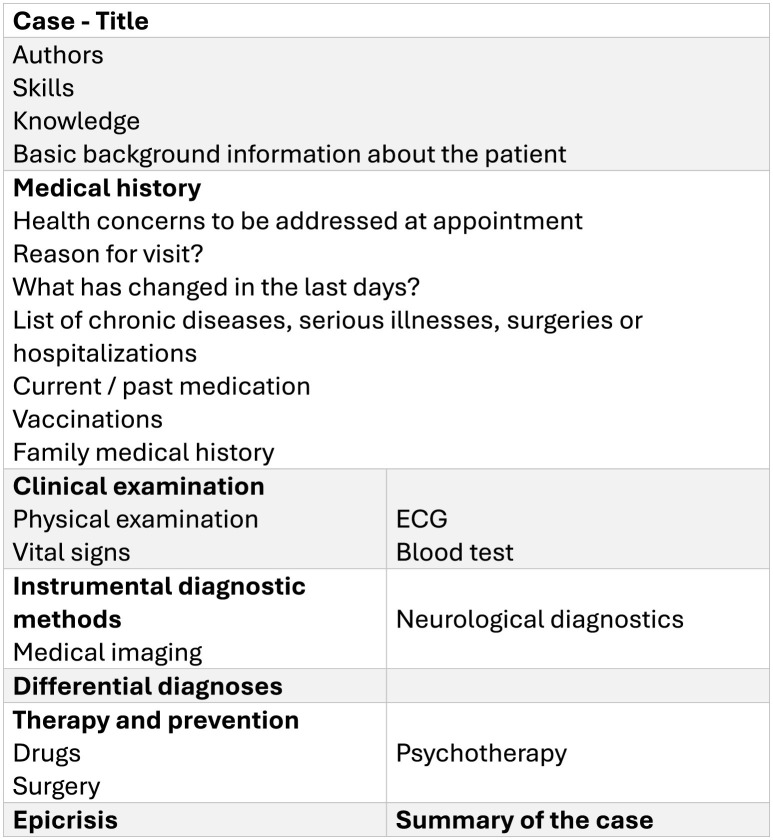
Course design adapted from Ertl et al. (13).

The course structure (Figure 2) (12, 13), case designs (Figure 3) (13), blueprints of patient cases (13), scientific background (22), preliminary evaluation results (14), consumer demand and satisfaction, course efficacy, and didactic background had already been described (12, 26, 28). The content was carried out in German. The main idea behind the course structure was to create a stimulating environment where students can apply their knowledge and learn from mistakes without fear of failure (29).

The concept for this course was based on Bloom's taxonomy criteria (30) and followed the CanMEDS framework (31) and aimed to foster interdisciplinary and crosslinked thinking (22). Case structure is interdisciplinary as they are real world cases. Observing and supporting students are vital strategies for fostering self-assessed learning, and interdisciplinary question-based blended cases promote individual learning plans for optimal knowledge acquisition. However, evaluating these newly implemented teaching concepts should enable further improvements in the medical curriculum. In total, 25,000 exam results were tracked; participants of the elective course were matched to students of the academic year for an individual matching (11, 26).

Beginning in 2013 with small groups, large student cohorts (*N* = 740 per academic year) have been involved since 2016, as the course can be utilized for preparation and training for the annual final examinations.

Various studies have already analyzed aspects such as student satisfaction, skills transfer, and the transition from declarative to procedural knowledge (7). Different forms of e-learning have been implemented in the medical curriculum, such as clinical rounds (elective non-structured discussion groups) in the fifth academic year (32). As clinical reasoning is the main task and important skill to be acquired before going to real-world care working with real patients in hospital in the sixth and last academic year, the “clinical practical year”, it is necessary to test, if at the end of the fifth academic year students perform well-enough all in all in the summative integrative exam (SIP 5). In this last exam, many questions refer to clinical reasoning. The structured e-learning approach of this course was used to reach students affectively and with more sustainability.

### 2.2 Exams

The “Summative Integrative Prüfung” (SIP), a summative integral exam, takes place every year/semester. The last exam (SIP 5) is particularly noteworthy, as it is conducted before the clinical internships. Additionally, the “Objective Structured Clinical Examination” (OSCE) takes place in the fourth academic year (33, 34). The OSCE is used after 4 years of intensive training to evaluate cardiopulmonary resuscitation, basic ultrasound, physical examination, diagnosis and history taking, communication with severe cases and difficult situations, and hospital emergency skills (35).

### 2.3 Participants

Medical students from all academic years (*n* = 5,800) were allowed to participate in the course from the winter term of 2016/17 to the winter term of 2020/21. There were no advertisements for the course, it has been added into the teaching catalog of elective offerings at the administrative platform of the university.

Students provided informed consent to participate and completed the elective course successfully. Subsequently, they then voluntarily submitted detailed questionnaires and exam results through a native built-in Moodle plugin that anonymized all data automatically.

A total of 296 medical students participated in the case-based teaching course and completed all the tasks (26); 32 co-registered students from different universities attended the course but were not analyzed. The study population comprised of 53% female and 47% male students with an average age of 24.1 ± 2.921 (median: 24.00). The nationality of the students was not recorded. From the entrance test a proportion of at least 75% Austrian, 20% foreign mainly German-speaking EU-countries, 5% non-EU countries is known, the language is German.

### 2.4 Questionnaire

The questionnaire assessed the educational environment and served as a secondary outcome measure. The instrument employed closed-ended questions, Likert scales, and multiple-choice items to evaluate various soft skills, as depicted in **Table 2** (questions in the first column). To capture the attitude toward the course the semantic differential, a standardized and validated assessment was used (results also in 13). To grasp affective involvement and attitudes toward a topic, a semantic differential technique is optimal as it also shows unconscious connotations. The questions presented in **Table 2** were self-selected with pending validation (26).

Apart from the main question (primary research question; exam grades), all information was descriptively analyzed and published in various studies (11–14, 22, 26).

### 2.5 Statistics

The data were collected from the Moodle website and transferred to an SPSS database. Students' answers were used to calculate general questions and explorative analyses. The exam results of the participants were analyzed and compared with those of all medical students. *T*-tests (for independent samples) for each cohort and exam were performed, and descriptive statistics were used for the questionnaire.

Due to multiple testing, a Bonferroni correction was performed (36). Numeric data are presented as mean ± standard deviation. Statistical analysis, graphics, and diagram generation were performed using SPSS^®^ 26.0 statistics software [SPSS Inc., Chicago, IL, USA (37)]. Bonferroni correction was applied for multiple comparisons, and statistical significance was assumed at a *p*-value < 0.01.

## 3 Results

### 3.1 Exam results

In Table 1 an overview of all exam results and group comparison between students with or without participation in the e-learning course are given. Participants in the e-learning course performed significantly better on the fourth (SIP4a), fifth (SIP5) (*p*-value < 0.01), and third years' exams (SIP 3) (*p* < 0.05) (Table 1).

**Table 1:** Exam results.

**Exam**	**Mean elective course participants**	**Mean control group**	**95% confidence interval**	**Significance**
SIP1a	Not applicable	2.99	Not applicable	Not applicable
SIP1b	2.0	2.75	−1.657 to 3.155	0.542
SIP2	1.5	2.11	−0.509 to 1.735	0.284
*SIP3*	*2.21*	*2.58*	*0.091 to 0.651*	0.012^*^
*SIP4a*	*2.08*	*2.42*	*0.207 to 0.484*	0.000^**^
*SIP5*	*2.36*	*3.2*	*0.410 to 1.273*	0.001^**^
OSCE	1.74	1.97	−0.65 to 0.536	0.124

^**^p-value < 0.01; ^*^p-value < 0.05.

No statistically significant effect on examination outcomes was observed for OSCE and the annual examinations in the first and second years (SIP1a, SIP1b, and SIP2).

Students in the e-learning course obtained significantly better grades, depending on their experience level (11).

### 3.2 Evaluation of the course and secondary outcome parameters

Secondary outcome parameters are presented in Table 2, including students' self-assessment (self-awareness) of soft skills and their attitude toward the offered course, which were evaluated using the open-response sections of the questionnaire employed in the study and have already been published (13, 22), but some notable parameters should be mentioned:

90.5% of the students [1.51 ± 0.774 (M ± SD)] recommended the elective course, and 74.7% suggested using a case-based teaching approach even for postgraduate board certification exams. Among the students, 65.2% preferred a bilingual teaching approach. The elective course was available only in Germany. Skill and exam tracking systems are implemented at many universities, and 56.1% of the students also favored such systems in our study.

**Table 2:** Attitude: questionnaire and results.

**Question**	**Answers**	**Results**
Do you want an accompanying case-based e-learning program to your normal course?	i Yes (+2) ii Rather yes (+1) iii Uncertain (0) iv Rather no (−1) v No (−2)	1.26 ± 0.896 (M ± SD)
Would you recommend this elective course?	i Yes (+2) ii Rather yes (+1) iii Uncertain (0) iv Rather no (−1) v No (−2)	1.51 ± 0.774 (M ± SD)
Would you use this elective course if it would cost a specific amount of money?	i No payment ii Monthly 15€ iii Monthly 10€ iv Monthly 5€ v One-off payment of 50–75€ vi One-off payment of 25–50€ vii One-off payment of 20€	57.9% would not pay 22.3% would prefer a one-off payment of 20€
Which language would you prefer?	i German ii English iii German and English	German: 32.3% English: 2.4% German and English: 65.2%
For which exam preparation would you suggest this elective course?		74.7%: board certification 25.3%: USMLE or IMPP 34.1%: no suggestion
Do you appreciate an exam tracking system within the elective course?	i Yes (+2) ii Rather yes (+1) iii Uncertain (0) iv Rather no (−1) v No (−2)	1.36 ± 0.852 (M ± SD)
My patient-centered view increased.	i Yes (+2) ii Rather yes (+1) iii Uncertain (0) iv Rather no (−1) v No (−2)	0.62 ± 0.928 (M ± SD)
Did my interdisciplinary thinking increase?	i Yes (+2) ii Rather yes (+1) iii Uncertain (0) iv Rather no (−1) v No (−2)	0.94 ± 0.888 (M ± SD)
Was my autonomous learning supported?	i Yes (+2) ii Rather yes (+1) iii Uncertain (0) iv Rather no (−1) v No (−2)	0.96 ± 0.994 (M ± SD)
Did my diagnostic skills improve?	i Yes (+2) ii Rather yes (+1) iii Uncertain (0) ic Rather no (−1) v No (−2)	1.02 ± 0.851 (M ± SD)
Did my argumentation competence for decision-making processes strengthen?	i Yes (+2) ii Rather yes (+1) iii Uncertain (0) iv Rather no (−1) v No (−2)	0.47 ± 0.970 (M ± SD)
Did my health literacy skills improve?	i Yes (+2) ii Rather yes (+1) iii Uncertain (0) iv Rather no (−1) v No (−2)	0.27 ± 0.954 (M ± SD)
After participating in this elective course, are you using economic considerations in your decision-making?	iYes (+2) ii Rather yes (+1) iii Uncertain (0) iv Rather no (−1) v No (−2)	0.24 ± 1.043 (M ± SD)
Do I question every decision-making after participating in this elective course?	i Yes (+2) ii Rather yes (+1) iii Uncertain (0) iv Rather no (−1) v No (−2)	1.28 ± 0.701 (M ± SD)
Would you recommend this elective course for your specialist medical training?	iYes (+2) • Rather yes (+1) iii Uncertain (0) iv Rather no (−1) v No (−2)	0.14 ± 0.922 (M ± SD)
Did you learn to re-evaluate and question yourself about the instrumental diagnosis being used?	i Yes (+2) ii Rather yes (+1) iii Uncertain (0) iv Rather no (−1) v No (−2)	0.84 ± 0.848 (M ± SD)
Have you learned to cope with unfamiliar situations better?	i Yes (+2) ii Rather yes (+1) iii Uncertain (0) iv Rather no (−1) v No (−2)	0.60 ± 0.913 (M ± SD)
Are you satisfied with the usability?	i Yes (+2) ii Rather yes (+1) iii Uncertain (0) iv Rather no (−1) v No (−2)	0.23 ± 1.288 (M ± SD)
Has collaborative learning been enhanced by the elective course?	i Yes (+2) ii Rather yes (+1) iii Uncertain (0) iv Rather no (−1) v No (−2)	−0.42 ± 1.209 (M ± SD)
Please tell us the size of your learning group.		1.65 ± 2.099 (M ± SD)

©Ertl (26).

A slightly positive increase in subjectively observed patient-centered thinking was reported [0.62 ± 0.928 (M ± SD)]. Furthermore, 60.6% of the students reported that they believed interdisciplinary thinking was cultivated [0.94 ± 0.888 (M ± SD)].

We expected the students to use the courses by themselves. Surprisingly, the students worked in groups to solve the cases, and the average group size was 1.65 students [1.65 ±2.099 (M ± SD)].

## 4 Discussion

Our research demonstrates the substantial impact on clinical reasoning of a structured case-based e-learning approach on students' exam performance, as evidenced by the statistically significant improvement in mean scores.

To our knowledge, no previous evaluation utilizing objective outcome measures such as examination grades has been conducted on a cohort of this magnitude.

From a legal perspective, it is not feasible to implement randomization in a mandatory examination at a public university.

The course was evaluated positively, affectively involving, as the detailed analysis of the semantic differential displaying attitudes, in general, had shown (13, 22).

Being affectively involved fosters intuitive 'fast thinking' (Kahneman) processes, which are necessary for a clinician to build a sound working alliance with the patient. The latter competency is hard to incorporate into training via algorithms or artificial intelligence (AI)-driven programs.

Additionally, slow thinking processes, that is conscious analytical processing, are trained via answering questions and getting feedback. Both ways of learning result in mentalized affectivity, which could be demonstrated as the training course stimulated inquiries and curiosity. Interdisciplinary collaboration was also stimulated. We implemented a forum to make discussions possible directly on the platform (13).

The Viennese e-learning platform, combined with simulated patient contact and an integrated feedback system, is designed to train crosslinking thinking in recognizing clinical patterns and arguing hypotheses and treatment decisions (10, 25). Moreover, controlled feedback is associated with student satisfaction and can be regarded as a major part of burnout prevention in medical students (7, 16, 23, 28).

As individual concepts for learning are required, teaching environments should also be augmented by traditional methods, such as continuous knowledge tests and skills training (e.g., following and practicing with the same cases as provided in the e-learning course via simulated patients) to achieve sufficient medical skills (38, 39).

Moreover, case-based teaching scenarios enriched with modern visualization software, artificial intelligence, and simulation programs should be fostered to train resident physicians or students alongside their bedside teaching. Precise feedback from experienced clinical mentors is a key feature for further curricular planning and, of course, for evaluation and research.

A limitation of the study design was the non-compulsory nature of the elective course. Another potential limitation of this study is the possibility of selection bias among highly motivated and skilled students who enrolled in the offered elective course for additional examination preparation. However, previous studies have similar limitations (2, 39–43). For the SIP1a, SIP1b and the OSCE (first academic year, first semester, and second semester), data available to perform the analysis and compare with the control group was insufficient. Nevertheless, based on our previous studies, we already gained significant results, particularly for the OSCE (7, 27).

A strength of this study is the comparison of the students at different levels. Case-based learning is essential for medical students at a certain training level. A consistent difficulty level of exams was ensured (35, 37, 44) because the exam and test levels were accurately aligned to a specific learning objective and competence level (CanMEDS) (31). Therefore, different annual examinations could be compared, variations in the examination performance of individual cohorts, therefore, need not be considered.

For further teaching considerations, carefully structured learning formats are essential, as medical knowledge expands at an unprecedented rate, and the clinician must be trained as an efficient, judicious, responsible decision-maker and compassionate professional (45).

To the best of our knowledge, this study analyzed the largest cohort of medical students.

In conclusion, the Viennese concept of interdisciplinary case-based e-learning combined with simulated patient contact and a feedback system on implemented questions significantly improves students' academic performance. Further development of the e-learning software, specifically artificial intelligence support for generating feedback questions, is warranted.

Nevertheless, the development of patient cases by clinical physicians remains a crucial component in imparting practical experience.

## Data Availability

The datasets presented in this study can be found in online repositories. The names of the repository/repositories and accession number(s) can be found below: Moodle platform.
